# Arginine-vasopressin in catecholamine-refractory septic versus non-septic shock in extremely low birth weight infants with acute renal injury

**DOI:** 10.1186/cc4917

**Published:** 2006-05-05

**Authors:** Sascha Meyer, Sven Gottschling, Ali Baghai, Donald Wurm, Ludwig Gortner

**Affiliations:** 1 Department of Neonatology and Pediatric Intensive Care, University Children's Hospital of Saarland, 66421 Homburg, Germany

## Abstract

**Introduction:**

The aim of this study was to assess the efficacy of arginine-vasopressin (AVP) as a rescue therapy in catecholamine-refractory septic and non-septic shock in extremely low birth weight (ELBW) infants with acute renal injury.

**Methods:**

Prospective assessment of AVP therapy in three ELBW infants with catecholamine-refractory septic shock and acute renal injury (mean birth weight 600 ± 30 g) and three ELBW infants with non-septic shock and acute renal injury (mean birth weight 770 ± 110 g) at a University hospital. The main outcome measures were restoration of blood pressure with adequate organ perfusion and survival at discharge.

**Results:**

In all three ELBW infants with catecholamine-resistant septic shock, systemic arterial blood pressure increased substantively with restoration of urine output after AVP administration (dosage, 0.035 to 0.36 U/kg/h; length, 70 ± 21 hours). In the three ELBW infants with non-septic shock, only a transient stabilization in mean arterial pressure with restoration of urine output was observed after AVP therapy (dosage, 0.01 to 0.36 U/kg/h; length, 30 ± 16 hours). The mortality rate was 1/3 in the sepsis group versus 3/3 in the non-septic group.

**Conclusion:**

AVP may be a promising rescue therapy in catecholamine-resistant shock in ELBW infants with acute renal injury. Larger prospective clinical trials are warranted to assess the efficacy and safety of AVP as a pressor adjunct in septic versus non-septic shock in ELBW infants.

## Introduction

Hypotensive, catecholamine-refractory shock is an important cause of morbidity and mortality in critically ill neonates. There is general agreement that there is depressed vasoconstrictor sensitivity to catecholamines in septic shock that can lead to vasodilatation and severe hypotension. Concentrations of vasopressin in plasma are significantly depressed in sepsis while vasopressin secretion is commonly increased in cardiogenic shock [1]. Clinical data indicate that a low serum vasopressin/norepinephrine ratio can predict impending septic shock in adults [2]. Recent clinical studies demonstrated that arginine-vasopressin (AVP) administration is most beneficial in septic patients [3-9]. However, AVP may also be employed successfully in children with states of depressed cardiac function [10].

AVP acts via vascular V1 receptors and renal tubular V2 receptors. V1 receptor stimulation leads to arterial vasoconstriction, and V2 stimulation increases renal free water re-absorption. Although no human data are available on V1 and V2 receptor mechanisms in pre-terms, animal studies demonstrated that the V1-receptor contributes to renal and cardiovascular responses to exogenous AVP *in utero *at the last third of gestation [11,12]. Here, we communicate our experience with AVP as a rescue therapy in six extremely low birth weight (ELBW) infants with catecholamine-refractory shock (three septic, three non-septic) and acute renal injury whose hypotension had not responded to prior fluid resuscitation, hydrocortisone therapy and high-dose catecholamine infusion.

## Materials and methods

This study was performed at the Department of Neonatology and Paediatric Intensive Care, University Children's Hospital of Saarland, and was conducted in accordance with the policy of our Institutional Review Board and the Helsinki Declaration. Between February 2004 and November 2005, ELBW infants (≤ 1,000 g birth weight) with catecholamine-resistant septic or non-septic shock and acute renal injury were consecutively enrolled.

Definitions of sepsis and septic shock were based on those established by the Society of Critical Care Medicine consensus conference of 1992 and its revised version published in 2003 with modification for normal values in neonates [13,14]. Non-septic shock was defined as cardio-circulatory failure with concomitant organ dysfunction (renal injury, hyperlactataemia) without an infectious etiology. Low cardiac output was defined as a shortening fraction ≤ 25%. Acute renal injury was based on the RIFLE classification, and included two criteria: glomerular filtration rate (two fold increase in serum creatinine) or urine output <0.5 ml/kg/h for at least six hours [15].

To maintain adequate systemic perfusion, all infants received norepinephrine (NE) and epinephrine (E) in a dose-up manner according to clinical judgements specific to each case, adequate volume resuscitation and hydrocortisone. Diuretic medication consisted of furosemide in varying dosage (0.5 to 2 mg/kg/h). AVP medication was started when patients developed catecholamine-resistant hypotension with inadequate tissue perfusion as demonstrated by acute renal injury and hyperlactataemia (>3 mmol/l). The AVP target dose was 0.01 to 0.12 U/kg/h. The dosage was adjusted according to the clinical course and included AVP bolus if the mean arterial blood pressure (MAP) was < 20 mmHg. After restoration of MAP and urine output, tapering of AVP was attempted.

Stenosis of the renal artery, renal vein thrombosis and post-renal causes for renal injury were excluded in all infants by ultrasonography. Serial echocardiography was performed in all infants to assess left ventricular function. All infants had an arterial line in place for invasive monitoring of arterial blood pressure. Daily laboratory monitoring included arterial blood gas analyses, serum lactate, complete blood count, serum chemistry and microbiological testing for infectious agents (bacterial, fungal, viral) as indicated.

Exclusion criteria to AVP administration included genetic disorders, malformations and diseases incompatible with life, birth weight and weight when included into this study > 1,000 g, sustained cardio-circulatory function by catecholamine administration, uncontrolled haemorrhage, prior hypersensitivity reaction to any constituent of AVP and failure to obtain parental informed consent. Infants with stenosis of the renal artery, renal vein thrombosis and post-renal causes of acute renal injury were also excluded as were infants with cardio-circulatory failure caused by an underlying cardiac pathology that required specific surgical intervention.

Main outcome measures were restoration of blood pressure with adequate organ perfusion and survival at discharge.

## Results

Between February 2004 and November 2005 a total of six ELBW infants with catecholamine-resistant septic (two bacterial and one fungal infection) and non-septic shock (two cardiogenic and one circulatory failure secondary to primary disease) and acute renal injury were consecutively enrolled in this study. All infants completed the study protocol. Demographic and clinical details are summarized in Table 1.

AVP dosage was comparable between septic (0.035 to 0.36 U/kg/h) and non-septic (0.01 to 0.36 U/kg/h) infants. Infant 1 was given an initial bolus of AVP (0.025 U) because of severe hypotension (MAP < 20 mmHg). The overall length of AVP administration was 70 ± 21 hours in infants with sepsis versus 30 ± 16 hours in non-septic infants. These differences are due to the early deaths of two twins with cardiogenic shock

**Figure 1:**
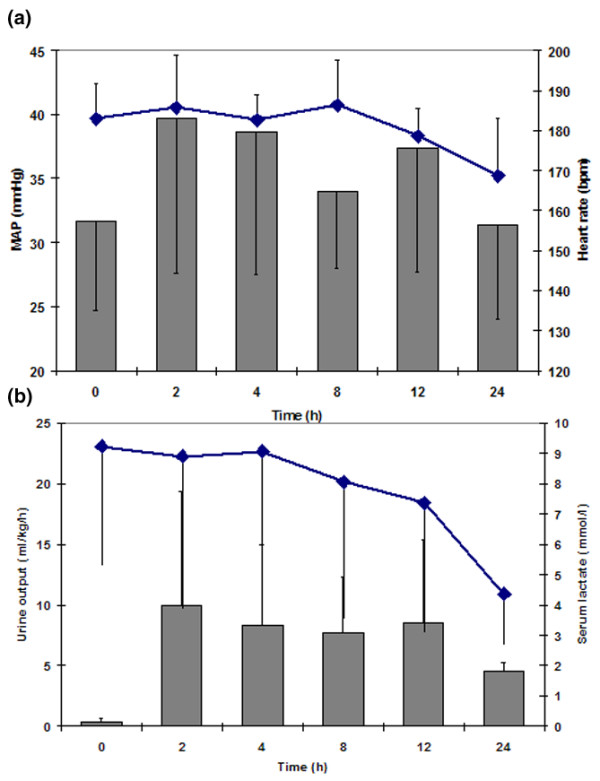
Cardiovascular parameters and urine output and serum lactate in ELBW infants with sepsis before and after initiation of arginine-vasopressin therapy. **(a) **Cardiovascular parameters: columns show mean arterial blood pressure (MAP; mmHg); lines show heart rate (beats per minute). Values given as mean ± standard deviation. **(b) **Urine output and serum lactate: columns show urine output (ml/kg body weight/h); lines show serum lactate (mmol/l). Values given as mean ± standard deviation.

**Figure 2:**
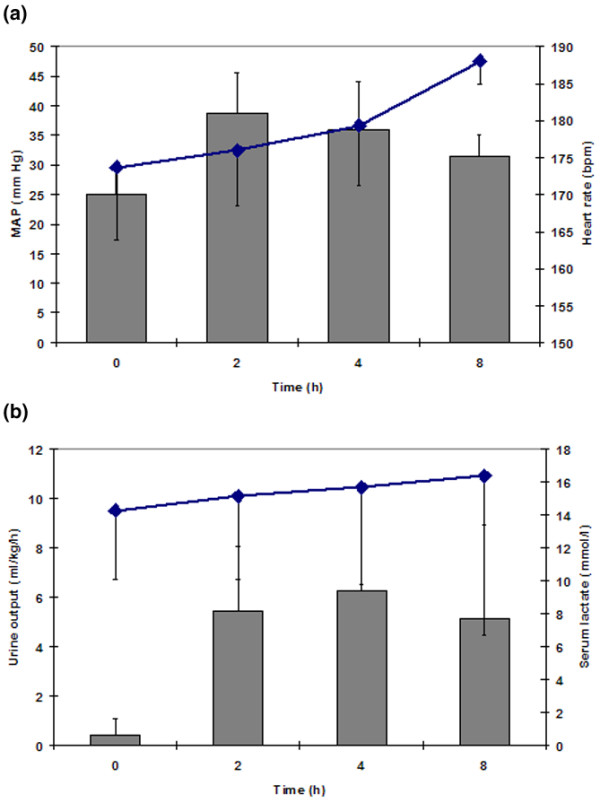
Cardiovascular parameters and urine output and serum lactate in ELBW infants with sepsis before and after initiation of arginine-vasopressin therapy. **(a) **Cardiovascular parameters: columns show mean arterial blood pressure (MAP; mmHg); lines show heart rate (beats per minute). Values given as mean ± standard deviation. **(b) **Urine output and serum lactate: columns show urine output (ml/kg body weight/h); lines show serum lactate (mmol/l). Values given as mean ± standard deviation.

In all six infants, MAP substantially increased within two hours after AVP administration (Figures 1a and 2a). In infants with septic shock, the increase in MAP was paralleled by a moderate decrease in heart rate, while in non-septic shock, the heart rate increased (Figures 1a and 2a).

At the beginning of AVP medication, all six infants were oligo-anuric. In parallel with the rise in MAP, two hours after starting AVP urine output increased substantially in all six infants (Figures 1b and 2b). However, the rise in urine output was not as pronounced in the two twins with cardiogenic shock (approximately 3 ml/kg/h). Following restoration of MAP, a pronounced decrease in serum lactate was seen in infants 1 and 2 with septic shock while it remained unchanged in infant 3. On the contrary, serum lactate continued to increase despite AVP in the two twins with cardiogenic shock. In infant 6, a transient, non-sustained decrease in serum lactate concentration was noticed.

Possible adverse effects related to AVP medication are detailed in Table 1. No acute side effects were seen (for example, digital and splanchnic hypoperfusion, abdominal distension, bloody stools, necrotizing enterocolitis), or myocardial ischemia, or worsening of metabolic/lactic acidosis that could be related to AVP administration.

The mortality rate was 1/3 in infants with sepsis-induced catecholamine-refractory shock compared to 3/3 in non-septic shock infants.

## Discussion

As reported in previous studies in children and adults [3-10], we demonstrated that AVP raised blood pressure in both septic and non-septic infants that was resistant to catecholamines (Figures 1a and 2a). Following restoration of tissue perfusion, a substantial increase in urine output was seen, which is in accordance with recent reports in children and adults with septic shock [3,9,16]. In our study, cardiovascular and renal changes induced by AVP were more pronounced and sustained in infants with septic shock, and associated with a fall in serum lactate (Figure 1b). The mortality rate in this group was 1/3. On the contrary, in non-septic infants, only a transient stabilization in cardiovascular and renal function could be achieved (Figure 2a,b). AVP administration did not have an impact on the poor prognosis of the three infants with non-septic catecholamine-resistant hypotension (mortality rate 3/3). The difference in survival rates between septic and non-septic infants cannot be related to the gestational age, birth weight or APGAR score. At the time of starting AVP, however, the three ELBW infants with non-septic catecholamine-resistant shock were in poorer clinical condition as shown by substantially higher serum lactate concentrations and the need for excessive catecholamines (Table 1).

There is still no clear concept of when to start VPA therapy in catecholamine-resistant (septic) shock. Recently, a large clinical study in adults with septic shock demonstrated the beneficial effects of initiating AVP therapy before NE requirements exceed 0.6 μg/kg/minute [17]. This is in accordance with our data, as the two surviving infants received NE and E in a dosage <0.6 μg/kg/minute prior to AVP medication (Table 1). Interestingly, a recent study in animals demonstrated that the combined infusion of NE and AVP improves hemodynamic variables compared with NE alone during sepsis, but not during cardiopulmonary resuscitation [18].

The differential effect of AVP can be related in part to its depletion in septic shock patients with hypersensitivity to exogenous AVP, whereas endogenous AVP release is increased in cardiogenic shock, causing a decreased response to exogenous AVP [1]. A low plasma AVP/NE ratio appears to be useful in predicting septic shock in adults [2]. In a recent study in children with meningococcal septic shock, however, AVP admission levels were appropriately elevated [19]. As we did not measure AVP serum levels, the above suggested mechanisms remain somewhat speculative. However, the prior administration of steroids in our study cohort might have affected endogenous AVP levels because cortisol suppresses the secretion of AVP in certain conditions [20]. Another limitation is the fact that systemic vascular resistances could not be determined in our ELBW infants, and thus it cannot be concluded with certainty that refractory shock was associated with vasoparalysis.

In most pediatric and adult clinical trials that assessed the efficacy of AVP in septic shock, terlipressin, an analogue of AVP with a longer duration of action (half-life of six hours versus six minutes for AVP), was given intermittently and not as a continuous infusion [4,7,21]. As hemodynamic profiles may change rapidly in children with septic shock – that is, transformation from hyperdynamic to hypodynamic shock with high systemic vascular resistance [21] – the use of AVP with a shorter time of action seems more appropriate. In one study in children with vasodilatory shock after cardiac surgery, AVP dosage ranged from 0.018 U/kg/h to 0.12 U/kg/h [10]. In another study in adults with vasodilatory septic shock, AVP was given at a rate of 2.4 U/h independent of body weight [6]. In our patients, AVP was administered as a continuous infusion, and titrated to the dosage that restored MAP and renal excretory function. In four infants, the mean dosage was in accordance with the above listed reports; AVP dosage escalation, which was in excess of standard dosage, was necessary in only two infants (non-survivors).

Major side effects of concern associated with AVP therapy are tissue hypoperfusion (mainly splanchnic) and a rebound phenomenon in vascular hyporeactivity with recurrent arterial hypotension [21,22]. No immediate side effects were seen in the surviving infants. In one infant (patient 6), substantial tissue liver necrosis was seen on autopsy, which could be related to prolonged AVP medication. With NE and E medication being continued after cessation of AVP, no rebound of clinical significance in arterial hypotension was noticed in our study cohort.

## Conclusion

This report adds further clinical experience on the use of AVP in catecholamine-refractory shock, indicating that it is also efficacious in ELBW infants. AVP may be a viable rescue therapy for ELBW infants in a refractory vasodilatatory state and acute renal injury when conventional therapies fail. To delineate the role of AVP in catecholamine-resistant shock in ELBW infants, further assessment of AVP safety and efficacy as a pressor adjunct in septic versus non-septic shock is warranted.

## Key messages

• 	AVP may be a viable rescue therapy for ELBW infants with intractable vasodilatation and acute renal injury to improve systemic arterial blood pressure and restore urine output when conventional inotropics fail.

• 	Further evaluation of AVP in larger controlled clinical trials is warranted to assess its efficacy and safety in septic versus non-septic shock in ELBW infants.

## Abbreviations

AVP = arginine-vasopressin; E: Epinephrine; ELBW = extremely low birth weight infants; MAP = mean arterial blood pressure; NE = norepinephrine; PDA = persistant ductus arteriosus. 

## Competing interests

The authors declare that they have no competing interests.

## Authors' contributions

SM was responsible for the conception and study design and data acquisition and analysis. SG was involved in data interpretation and drafting the manuscript. AB was responsible for data acquisition and interpretion of data. DW was responsible for data acquisition and drafting the manuscript. LG was involved in data interpretation and drafting the manuscript.

## Figures and Tables

**Table 1:** Patient characteristics and clinical details

Patient	Age (gender)/birth weight/APGAR score	Underlying disease/treatment	Cause/time of onset of shock	Urine output/Increase in serum creatinine/Serum lactate prior to AVP	Echocardiography	Dosage/duration of AVP	NE/E prior to AVP	Further NE/E	Clinical outcome/complications
1	24 + 6 wks (F); caesarean delivery; 600 g APGAR: 7/9/9	RDS, PDA Mechanical ventilation Surgical closure of PDA	*Klebsiella pneumoniae *sepsis 10th day of life	0.2 ml/kg/h 2.3 times 8.5 mmol/l	SF: 34–38%	After an initial bolus of 0.025 U, 0.035 U/kg/h 36 hours	NE: 0.5 μg/kg/minute E: 0.5 μg/kg/minute	Continuation of NE/E over 28 hours after cessation of AVP therapy in decreasing dosage	Survived; BPD; ROP II; Two cystic lesions (occipital and periventricular; 3–4 mm in diameter) most probably residues from intracranial hemorrhage
2	26 + 5 wks (F); caesarean delivery; 660 g APGAR: 3/7/8	RDS, PDA Mechanical ventilation Surgical closure of PDA	*Candida parapsilosis *sepsis 12th day of life	0.1 ml/kg/h 2.1 times 14.4 mmol/	SF: 33–36%	0.10 U/kg/h 118 hours	NE: 0.5 μg/kg/minute E: 0.5 μg/kg/minute	Continuation of NE/E over 20 h after cessation of AVP therapy in decreasing dosage	Survived; BPD; bilateral intraventricular hemorrhage without developing hydrocephalus; ROP I; no ischemic lesions secondary to AVP therapy
3	27 + 6 wks (M); caesarean delivery; 550 g APGAR: 6/7/7	RDS, prior acute renal injury possibly related to indomethacin administration Mechanical ventilation	*E. coli*/*Staph. epidermidis *sepsis 5th week of life	0.2 ml/kg/h 1.5 times 5.2 mmol/l	SF: 35–36%	Initially 0.12 U/kg/h, increased to 0.36/U/kg/h 85 hours	NE: 0.5–1.0 μg/kg/h E: 0.5–1.0 μg/kg/h	Continuation of NE/E over the next 6 days after cessation of AVP therapy in increasing dosages	Recurrent episode of acute renal injury; died; autopsy showed severe RDS; no ischemic lesions secondary to AVP therapy
4	Twin I: 26 + 1 wks (M); spontaneous vaginal delivery; 890 g APGAR: 4/7/8	RDS Progressive left ventricular dilatation Hyperkalemia Pneumothorax HFOV Drainage of pneumothoraces Intravenous calcium, β_2_-mimetics, insulin	Low-cardiac output failure 3rd day of life	0.2 ml/kg/h 2.0 times 14.9 mmol/l	SF: 15–20% 1st to 2nd degree mitral valve insufficiency PDA ruled out	Initially 0.01 U/kg/h, increased to 0.1 U/kg/h 21 hours	NE: 1.5 μg/kg/minute E: 1.5 μg/kg/minute	Despite AVP increased demand for catecholamines (NE/E: 3 μg/kg/minute)	Died after 21 hours of AVP therapy of cardio-respiratory failure; no ischemic lesions secondary to AVP therapy. A congenital cardiac malformation and cardiomyopathy were ruled out by autopsy
5	Twin II: 26+1 wks (M); spontaneous vaginal delivery; 880 g APGAR: 6/7/7	PIE Progressive left ventricular dilatation Hyperkalemia HFOV Intravenous calcium, β_2_-mimetics, insulin	Low-cardiac output failure 3rd day of life	0.4 ml/kg/h 2.2 times 20.0 mmol/l	SF: 15–20% 1st to 2nd degree mitral valve insufficiency PDA ruled out	Initially 0.01 U/kg/h, increased to 0.03 U/kg/h 8 hours	NE: 3.0 μg/kg/minute E: 3.0 μg/kg/minute Enoximone: 5 μg/kg/minute	Despite AVP increased demand for catecholamines (NE/E: 5 μg/kg/minute)	Died after 8 hours of AVP therapy of cardio-respiratory failure; no ischemic lesions secondary to AVP therapy. A congenital cardiac malformation and cardiomyopathy were ruled out by autopsy
6	Twin I: 24 + 5 wks (F); caesarean delivery; 550 g APGAR: 1/5/7	RDS Bilateral pneumothoraces Second degree intracranial hemorrhage Mechanical ventilation Drainage of pneumothoraces	Non-septic circulatory collapse secondary to primary disease 6th day of life	0.3 ml/kg/h 2.7 times 10.9 mmol/l	SF: 32–34%	Initially 0.12 U/kg/h, increased to 0.36 U/kg/h 61 hours	NE: 0.4 μg/kg/minute E: 0.4 μg/kg/minute	Despite AVP catecholamines (NE/E: 0.6–0.8 μg/kg/minute)	Died after 61 hours of AVP medication; liver tissue necrosis seen on autopsy as a possible sequelae of AVP medication

AVP, arginine-vasopressin; BPD: Bronchopulmonary dysplasia; E, epinephrine; F, female; HFOV, high frequency oscillatory ventilation; M, male; NE, norepinephrine; PDA, persistant ductus arteriosus; PIE, pulmonary interstitial emphysema; RDS, respiratory distress syndrome; ROP, retinopathy of prematurity; SF, shortening fraction.
